# Notes and News

**Published:** 1900-03-24

**Authors:** 


### NOTES AND NEWS.

HOSPITAL MEETINGS.

St. Mary's Children's Hospital, Plaistow.—Annual Meeting at half-past three p.m., on Thursday, March 22nd, at the Hospital.

West End Hospital, 73, Welbeck Street.—Annual Meeting at four p.m., on Friday, March 23rd.

St. Peter's Hospital, Henrietta Street, Covent Garden.—Annual Meeting at four p.m., on Tuesday, March 27th, at the Hospital.

North London Hospital for Consumption, Hampstead.—Annual Meeting at four p.m., on Wednesday, March 28th, at 41, Fitzroy Square.

Home, Hospital, and Association, Fitzroy Square, W.—Annual Meeting at four p.m., on Wednesday, March 28th.

Royal Eye Hospital, Southwark.—Annual Meeting at four p.m., on Wednesday, March 28th.

The report to be presented to the annual meeting of the London Polyclinic, which will take place next Wednesday, shows that this institution has made considerable progress. It is stated that the list of original subscribers had reached the satisfactory total of 607 at the end of last year, and that the classes, lectures, and consultations have been well attended.

An interesting meeting was held recently at Tunbridge Wells in aid of the League of Mercy. Dr. Collins, of the London County Council, and hon. secretary of the league, addressed the meeting. The formation of no less than ten centres in the neighbourhood was reported, showing an imposing list of officers who had enrolled their services on behalf of the league. Already £121 has been collected in the neighbourhood.

A stirring protest against hospital abuse was made recently by the Recorder at the Dublin City Sessions. A patient had been frequenting a hospital after injuries received in an accident, although he could well afford to pay for private treatment, being in receipt of £2 per week. "Such actions," the Recorder is reported to have said, "freed money for the publican's till. Disorder followed, and the people were poorly fed, and up jumped the death-rate to 50 in the thousand."

In the House of Commons on Saturday Sir W. Cameron Gull asked the First Lord of the Treasury whether, having regard to the representations made to Her Majesty's Government through the President of the Local Government Board, the Government would this session undertake legislation relieving hospitals in Great Britain from local rates; and Mr. Balfour replied that the Government had expressed sympathy with the object, but that the question was surrounded with considerable difficulty; and the best plan would be to have a Committee of the House to look into the subject.

Rather an amusing discussion took place at the recent annual meeting of the Huntingdon County Hospital. It arose out of a motion to alter the rule relating to the dismissal of patients for bad conduct. As the rule stood any patient who smoked or played cards was liable to instant dismissal, and the mover contended this was unreasonable severity. One speaker admitted the regulation savoured of the Middle Ages, but several objected to the toleration of card-playing lest it should foster gambling. Eventually it was decided to allow smoking under reasonable restrictions, and card-playing without money passing. It was subsequently revealed that the decision had been anticipated by the matron, who, oblivious to any rule on the subject, had permitted both smoking and card-playing at suitable hours. Her disregard of the rules having been unattended with evil consequences the governors decided to leave the conduct of the matter in her hands.

The managers of the Aberdeen Royal Infirmary have recently placed a contract for printing in the hands of the University Press of that city. This act has occasioned a remonstrance from the Scottish Typographical Association on the ground that the University Press is a non-union firm, insinuating that the choice was a prejudiced one on the part of the infirmary managers, and intimating that the association intended to institute a campaign against the Infirmary on account of their action. The treasurer of the infirmary sent a reasonable and moderate reply to the communication, pointing out that the only consideration which had influenced the management had been the interests of the infirmary, and that they were quite unaware of and unconcerned with any trade question. He further refused to believe that the workpeople of Aberdeen would be interested by personal and unworthy motives against an institution existing solely for the benefit of the sick poor. The question of local supply brought so prominently forward at Aberdeen is one that concerns all charitable institutions. At Aberdeen the infirmary management have acted in a disinterested and impartial manner in selecting the most suitable tender. This course should be pursued by all responsible for the expenditure of public monies for charitable purposes, but unfortunately this is not always the case, and in some instances inferior or expensive supplies are accepted as a sort of bribe or repayment to the local tradesmen in return for any interest they may take in the local institution. Thus the hands of those who would manage economically are tied, and charity is discounted by self-interest.

